# Development and validation of a prognostic model for esophageal carcinoma based on immune microenvironment using system bioinformatics

**DOI:** 10.1002/cam4.4985

**Published:** 2022-06-30

**Authors:** Chenchun Fu, Shicheng Feng, Sheng Wang, Xiangyu Su

**Affiliations:** ^1^ Department of Oncology Zhongda Hospital, Southeast University Nanjing China

**Keywords:** esophageal cancer, immunotherapy, prognosis, tumor microenvironment, tumor mutation burden

## Abstract

Esophageal cancer (EC) is an aggressive malignancy that accounts for numerous cancer‐related deaths worldwide. The multimodal combination therapy approach can be potentially used to treat EC effectively. However, distinct biomarker of significant specificity are still needed to develop individualized treatment strategies and provide accurate prognostic predictions. Therefore, we aimed to investigate the associated genes subtypes identified were, IFN‐γDominant, Inflammatory, Lymphocyte Depleted, etc. and construct a risk model based on these genes to predict the overall survival (OS) of patients suffering from EC. Three immune subtypes were defined in the Cancer Genome Atlas (TCGA) cohort with different tumor microenvironment (TME) and clinical outcomes based on radio‐differentiated immune genes. Subsequently, a risk model of immune characteristics included the immune cell infiltration levels and pathway activity was developed based on the genomic changes between the subtypes. In the TCGA dataset, as well as in subgroup analysis with different stages, gender, age, and pathological type, a high‐risk score was identified as an adverse factor for OS using the method of the univariate Cox regression analysis and tROC analysis. Furthermore, it was observed that the high‐risk group was characterized by depleted immunophenotype, active cell metabolism, and a high tumor mutation burden (TMB). The low‐risk group was characterized by high TME abundance and active immune function. Differences in the biological genotypes may account for the differences in the prognosis and treatment response. Extensive research was carried out, and the results revealed that the low‐risk group exhibited a significant level of therapeutic advantage in the field of immunotherapy. A risk model was developed based on the immune characteristics. It can be used to optimize risk stratification for patients suffering from EC. The results can potentially help provide new perspectives on treatment methods.

## Graphical abstract

In this study, three immune subtypes were defined in the Cancer Genome Atlas (TCGA) cohort with different tumor microenvironment (TME) and clinical outcomes based on radio‐differentiated immune genes. Subsequently, a risk model of immune characteristics included the immune cell infiltration levels and pathway activity was developed based on the genomic changes between the subtypes. As well as in subgroup analysis with different stages, gender, age, and pathological type, a high‐risk score was identified as an adverse factor for OS using the method of the univariate Cox regression analysis and tROC analysis. Furthermore, we observed the high‐risk group was characterized by depleted immunophenotype, active cell metabolism, and a high tumor mutation burden (TMB).

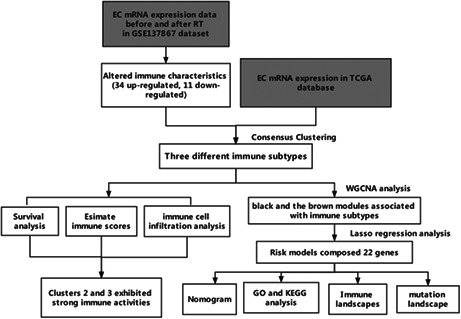

## 1 INTRODUCTION

Esophageal cancer (EC), an aggressive malignancy, has been identified as the eighth most common type of cancer globally.^1^ The progress made in the fields of neoadjuvant therapy and perioperative care has helped improve the overall survival rate of resectable EC cases significantly. However, the prognosis of individuals at an advanced stage of EC remains poor.^2^, ^3^, ^4^ The overall 5‐year survival rate is less than 13% after EC diagnosis, and this can be attributed to recurrence, significant invasion, and metastasis.^5^ Radiotherapy (RT) can be effectively used to treat patients who do not need to be subjected to surgical conditions and are suffering from locally advanced EC.^6^ The failure of the local RT treatment method can be attributed to radiotherapy resistance or tumor heterogeneity. It has been observed that persistent or recurrent disease is observed in 40%–60% of the patients suffering from EC who receive RT.^7^, ^8^ Thus, there is an urgent clinical need to identify biomarkers for EC treatment and prognosis prediction to provide better treatment options to patients with poor prognoses.

RT is an important component that affects the standard of care for cancer patients.^9^ RT can induce the damage of double‐stranded deoxyribonucleic acid (DNA) resulting in the death of tumor cells. This can be realized through various biological pathways such as necrosis, apoptosis, mitotic disaster, autophagy, or replicative aging.^10^, ^11^ Changes in the genome regulate the immune system and the tumor microenvironment (TME), and the positive regulation of RT on tumor immunity has contributed to the emergence of new combination treatment methods.^12^, ^13^ Tumor immune microenvironments are the promoting factors associated with carcinogenesis and the development process. Immunotherapy, in addition to surgical and radiation treatment methods, has emerged as a potential treatment method for numerous malignancies.^5^ Results from several preclinical trials have revealed that a combination of RT and immunotherapy can be used to improve the control of local and systemic tumors.^14^ The cancer survival rate can be prolonged, and the progress and development of cancer in preclinical tumor models can be delayed by combining RT with anti‐CTLA‐4,^15^ anti‐PD‐1,^16^ or anti‐PD‐L1 therapy methods.^17^, ^18^

However, mixed results are always obtained when the immunotherapy methods currently in practice are used to treat EC. This can be partly attributed to the lack of reliable markers that can predict treatment response.^19^ It is known that immune‐related genes (IRG) and tumor‐infiltrating immune cells play important roles in carcinogenesis and tumor progression.^20^ The complex interactions between infiltrating immune cells, immune cell cytokines, and the microenvironment of tumor cells dynamically regulate the incidence of EC.^21^ Although assessing TME status is a powerful method for prognostic assessment and drug response prediction, the components of TME are difficult to determine.^22^ As IRG is the basis of immune system function, the active regulation of RT on the IRG transcriptional map provides us with new perspectives on the treatment methods. The prognostic characteristics based on IRG can aid researchers in understanding TME in patients suffering from EC and contribute to the prognostic evaluation and individualized treatment strategies of EC.^23^

Here, the TME subtypes and clinical outcomes of three different immune landscapes were identified in the TCGA‐EC cohort depending on the changes in IRG (before and after radiotherapy). Clusters 2 and 3 were immune‐activated and are characterized by a good prognosis. Subsequently, an accurate model for predicting OS prognosis was constructed for EC patients based on the potential TME‐related prognostic genes. In addition, the model can characterize the immune landscape of the patients and predict the responses of the patients when they are subjected to conditions of chemotherapy and immunotherapy.

## 2 METHODS

### 2.1 Data collection

The GEO database (https://www.ncbi.nlm.nih.gov/geo/) was analyzed, and the GSE137867 dataset of the GPL15207 platform was obtained to identify the variations in the genomes of the EC patients. The variations occurring before and after the patients were subjected to conditions of neo‐chemoradiotherapy were recorded. The dataset contained matched four samples before treatment and four samples after treatment from four patients with esophageal squamous cell carcinoma.^24^ Following this, the dataset was standardized using the log2 function in the “limma” package for the exploration of the cohort. The RNA‐Seq data corresponding to EC mRNA expression patterns and the related clinical information of the patients were downloaded from the TCGA database (https://cancergenome.nih.gov/). In addition, 160 tumor samples were obtained after eliminating the participants for whom insufficient clinical information was obtained. The original Fragments per Kilobase per Million (FPKM) expression data were standardized to Transcripts per Kilobase per Million (TPM), and these were used for training the cohorts. The list of genes related to immune function was obtained from the ImmPort database (https://www.immport.org/resources),^25^ which contained 2496 immune‐related genes.

### 2.2 Identification of immune subtypes associated with RT

Differential immune‐related genes in the exploration cohort (before and after RT) were identified using the “limma” package. FDR < 0.05 and |fold change (FC) > 1| was considered the threshold to avoid omission. The differential immune genes were studied and analyzed, and based on the results, the hierarchical consensus clustering method was used to identify the immune subtypes in the TCGA cohort using “ConsensuClusterPlus” (R package).^26^ Based on Ward's linkage and Euclidean distance, we used the unsupervised clustering method “pam “to verify the consistency of these immune subtypes and modules. The calculation was repeated 1000 times to confirm the stability of the classification.

### 2.3 Assessment of immune activity

Marker genes for immune cell types were obtained from the paper reported by Bindea et al.^27^ Marker genes for immune activity pathways were obtained from previously reported papers.^28^ The specific gene sets that are considered are presented in Table S1. The single‐sample gene set enrichment analysis (ssGSEA) method was used to assess immune cell infiltration and immune pathway activity. The R‐package “GSVA” was used for the same. The ESTIMATE method was utilized to calculate the immunological activity score and tumor purity score of the samples.^29^

### 2.4 Development and validation of risk models

The TCGA cohort was used to generate the risk scores. First, the “limma” package was used to identify the differential genes among the subtypes, and | Log_2_ fold change (FC)| >1.0 was considered as the threshold. The FDR was <0.05. Next, a scale‐free co‐expression network was constructed using the R package “WGCNA”.^30^ Differentially expressed genes (DEGs) most related to immune subtypes were determined by analyzing the transcriptional data associated with the DEGs. An appropriate soft threshold (*β* = 6) was selected to confirm the scale‐free co‐expression network. Furthermore, modeling was done using genes from the two modules that were most positively and negatively linked to the immune subtype. The module genes that could be used for prognosis were detected following the univariate Cox regression analysis method. The Cox proportional risk model with LASSO penalty was used to select the risk model characterized by the best prognostic value for genes exhibiting prognostic potential, and the stability of the model was determined following the 10‐fold cross‐validation method. The randomness of the 10‐fold cross‐validation was taken into account, and 100 iterations were performed to determine the most stable optimal prognostic model. The best genetic models were used to establish the risk scores. The model was constructed based on the LASSO coefficients and mRNA expression. The formula used is as follows:

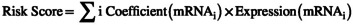

The “caret” package was used to randomly divide the entire TCGA cohort into two subgroups (1:1 ratio) to assess the accuracy of the model. The “survcomp” R package was used to determine the consistency C index, as reported by Harrell et al.^31^ The predictive performance of the model was validated across all three data sets. A high C‐index suggested that the model could be used to obtain highly accurate predictions.

### 2.5 Somatic mutations in the TCGA cohort

The corresponding Mutect2 platform mutation data were retrieved From the TCGA cohort using the “TCGAbiolinks” package.^32^ The cumulative amount of non‐synonymous mutations in the sample was first calculated to analyze the differences in mutation load in both categories. The genes characterized by the minimum mutation number > 10 were identified using the “maftools” R package, and the Chi‐square test was conducted to compare the differences in the gene mutation frequency among the two groups. MAFTools was used for visualization.^33^

### 2.6 Predicting the response of subtypes toward immunotherapy

The TIDE algorithm was initially used to predict the response of the patients toward anti‐PD1 and anti‐CTLA4 therapy methods to determine the predictive effect of the risk model on immunotherapy.^34^, ^35^ The unsupervised subclass mapping (https://cloud.genepattern.org/gp/) method was used^36^ to compare the high‐ and low‐risk subgroups and a published dataset of 47 patients who responded to PD1 and CTLA4 therapy^37^ to determine the response of the two subtypes toward immunotherapy. The cut‐off for significant or no response (toward anti‐PD1 and anti‐CTLA4 treatment methods) was set at an FDR value of <0.05. Furthermore, the independent dataset IMvigor210 was obtained and examined to assess the predictive efficacy of the risk score. Under the Creative Commons 3.0 license, the IMvigor210 dataset was downloaded from http://research‐pub.gene.com/IMvigor210CoreBiologies. Two hundred and ninety‐eight cases of urothelial carcinoma with complete medical data were analyzed.

### 2.7 Prediction of sensitivity toward chemotherapy and potential small molecule compounds

The Genomics of Drug Sensitivity in Cancer (GDSC) database^38^ was analyzed, and the five first‐line EC drugs (cis‐platinum, docetaxel, doxorubicin, paclitaxel, and 5‐fluorouracil) were selected using the R package “pRRophetic.” The ridge regression method was used to determine the 50% maximum inhibitory concentration (IC50) of each sample and determine the sensitivity of the two groups toward chemotherapy. The predictive accuracy was assessed following the process of 10‐fold cross‐validation. In addition, differential genes in both the subgroups were identified as novel treatment targets, and the CMap database (https://clue.io/) was analyzed to identify the compounds that could be potentially used to target these genes. Connectivity score and enrichment score range from 100 to −100. A positive score indicated that the compounds could cause or aggravate the state of the disease, and a negative score indicated that the compounds could alleviate or reverse the state of the disease.^39^ The drugs characterized by the enrichment fractions <−90 were identified as the small molecule compounds that could be potentially used for treatment.

### 2.8 Bioinformatics and statistical analyses

R software (Version 4.04) was used to perform the statistical analyses and mapping. R package (“clusterProfiler”) was used for functional annotation of genes. The Kruskal–Wallis test was conducted to study more than two groups, while the Wilcoxon test was conducted to compare two groups. The proportions were compared by conducting the Chi‐square test. The survival curves for subgroups in each dataset were generated using the Kaplan–Meier plotters. Logarithmic rank tests were conducted to assess the differences in statistical significance. The R package “survivalROC” was used for time‐dependent receiver operating characteristic (tROC) analysis to calculate the time‐dependent area under the curve (AUC) and evaluate the predictive ability of the variables. The R packages “survival” and “rms” were used for univariate and multivariate COX regression analyses and to generate nomogram and calibration curves, respectively. The DCA package was used for decision curve analysis (DCA).^40^ Unless otherwise specified, a two‐tailed statistical significance was represented by *p* < 0.05.

## 3 RESULTS

### 3.1 Identification of altered immune characteristics before and after RT

**FIGURE 1:**
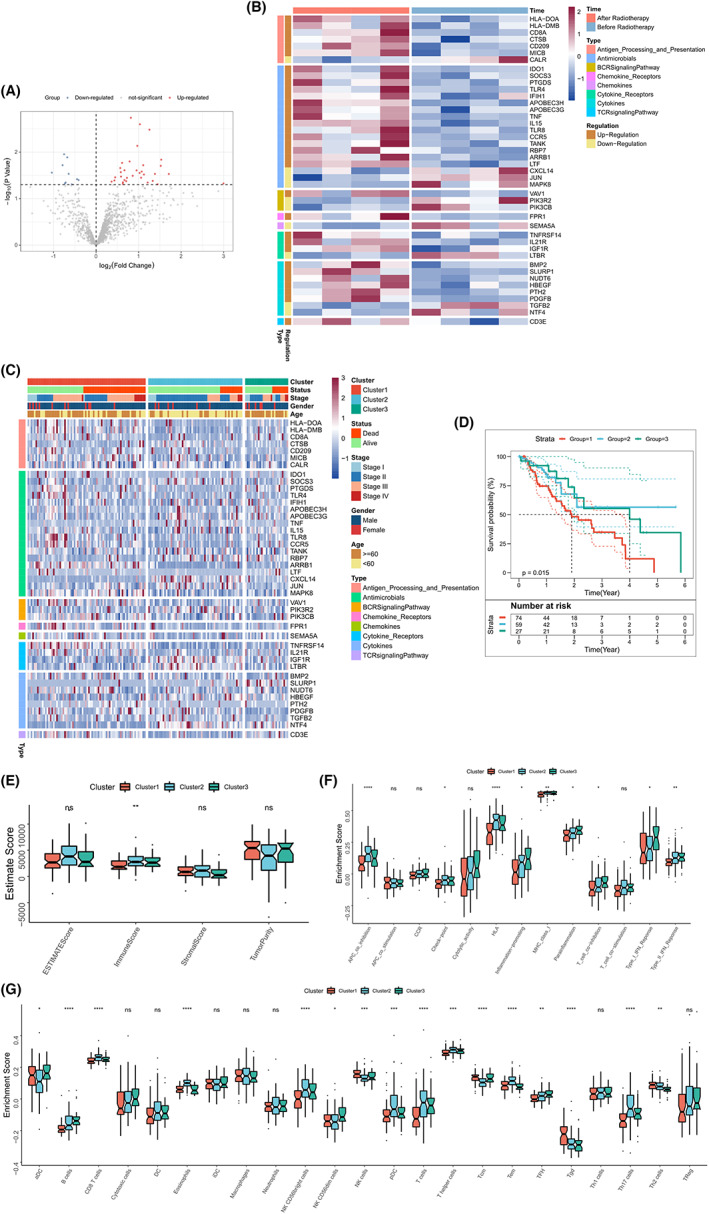
Immune subtypes based on differential IRG before and after radiotherapy in the TCGA‐EC cohort. (A) Volcano map presenting the differential IRG before and after radiotherapy in the GSE137867 cohort. (B) Heat map presenting the differential IRG before and after radiotherapy in the GSE137867 cohort. (C) Unsupervised clustering of differential IRGs in the TCGA cohort. Rows represent IRG, columns represent samples, and the clinical features of the patients are also marked. (D) Kaplan–Meier curves of OS in the patients with different immune subtypes. Log‐rank *p* = 0.015. (E–G) Box plots present the ESTIMATE score (E), immune pathway activity (F), and proportion of immune cell infiltration (G) for patients with three immune subtypes. *p < 0.05; **p < 0.01; ***p < 0.001; ****p < 0.0001.

The GSE137867 dataset was considered the discovery cohort to identify the altered immune characteristics before and after therapy. We identified 45 IRGs which exhibited changes when patients with EC were subjected to conditions of RT. A P value of <0.05 was the threshold (Figure 1A). There were 34 up‐regulated and 11 down‐regulated genes. Analysis of the heat maps revealed the transcriptional maps of differential IRGs (Figure 1B). The detailed results are presented in Table S2.

### 3.2 Identification of the immune subtypes of the EC samples based on immune characteristics

The ConsesusClusterPlus package associated with the R software was used for unsupervised clustering, and three different immune subtypes were identified according to the optimal cluster number *k* = 3 (Figure 1C; Figure S1A–F). Results from survival analysis revealed the poor prognosis of Cluster 1 patients. A good prognosis was realized for the patients belonging to Clusters 2 and 3 (Log Rank test, *p* < 0.0001; Figure 1D). The immune microenvironments of various subtypes were examined and compared to verify the inherent biological variations which contributed to the diversity of the clinical phenotypes. The results obtained using ESTIMATE revealed that patients belonging to Cluster 2 were characterized by high immune scores, and patients in Cluster 1 were characterized by the lowest immune scores. Although there was no difference in tumor purity among the three subgroups, patients belonging to Cluster 2 exhibited lower tumor purity scores compared to the others (*p* > 0.05; Figure 1E). Subsequently, the immune cell infiltration scores and pathway activity of the subgroups were quantified. Interestingly, the scores of contents related to antigen presentation, including DC, Pdc, HLA, and MHC class I, were high. The extents of infiltration of most of the cellular immune and antitumor‐related immune cells, such as CD8T cells B cells, TFH, T cells, TEM, and TH17, were significantly more in Clusters 2 and 3 than the extent of infiltration recorded for Cluster 1. High activity scores for Type I and Type II interferon pathways were recorded for Clusters 2 and 3 (*p* > 0.05; Figure 1F,G). These results suggested that Clusters 2 and 3 exhibited strong immune activities. They might exhibit stronger anti‐tumor responses (compared to Cluster 1), resulting in a better prognosis. Cluster 1 was characterized by low immunoactivity and high tumor purity, indicating the presence of a type of immunosuppressed tumor, resulting in a poor prognosis. In conclusion, immune subtypes are closely related to immune activation and immune microenvironment components, which may result in differences in prognosis.

### 3.3 Risk models associated with immune subtypes

**FIGURE 2:**
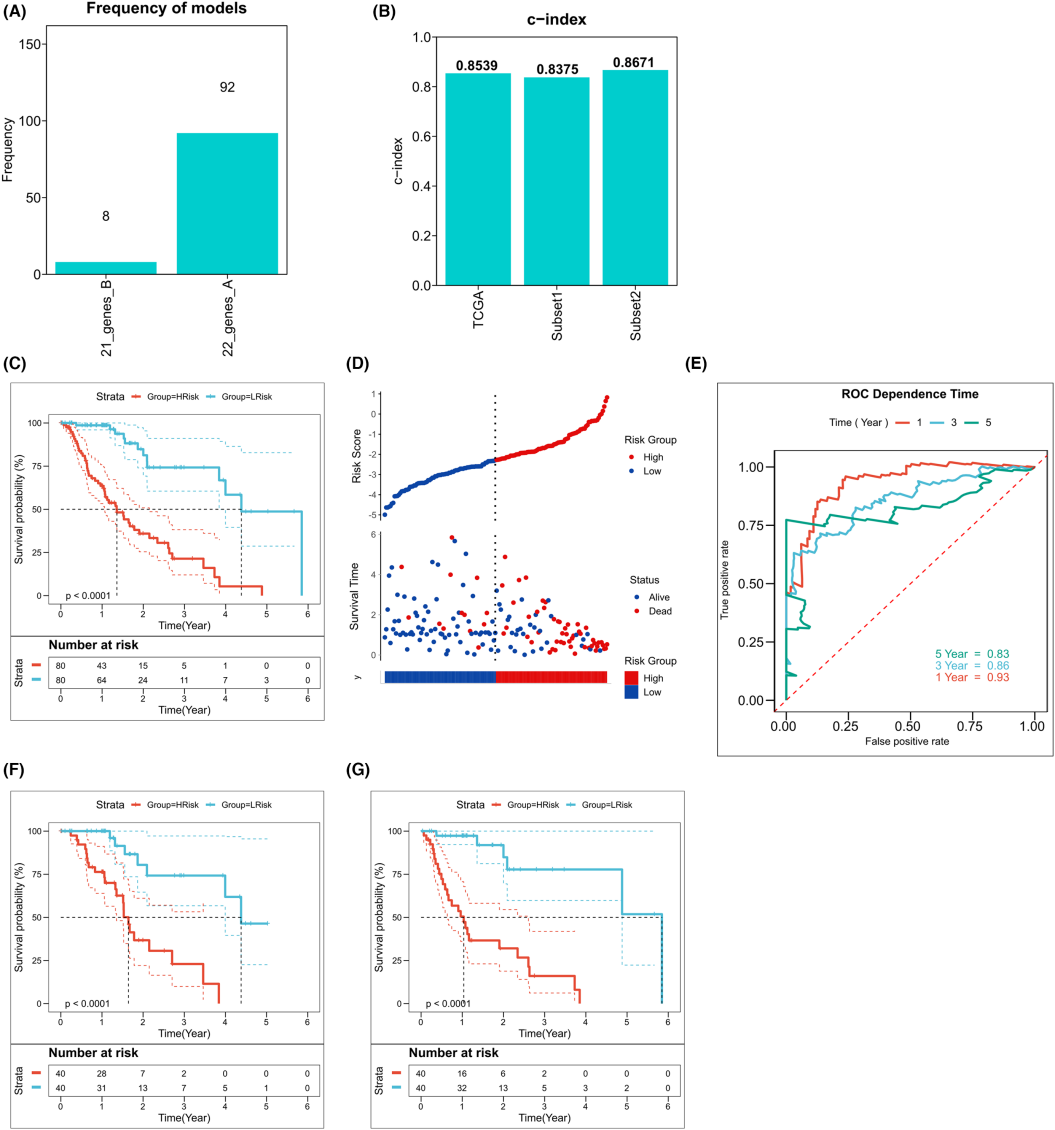
Construction of an immune characteristic risk model based on the immune subtypes. (A) Frequency of occurrence of different gene combinations in the LASSO Cox regression model. (B) Training and test sets' C indices. (C) Survival curve corresponding to the high‐low risk subgroup of the TCGA cohort. Log‐rank: *p* < 0.0001 (D) Survival status of the patients in the TCGA cohort with high and low risks scores. (E) Risk score ROC curves at 1, 3, and 5 years in the TCGA cohort. (F, G) Survival curves of the high and low‐risk sub‐groups in Subgroup 1 (F) and Subgroup 2 (G).

Differential genes among the subtypes were identified by taking into account the interesting biological differences among the three immune subtypes. The WGCNA analysis method was used for analysis (based on the transcriptional data obtained for differential genes and immune subtypes of samples) to further identify the genes most associated with immune subtypes. The appropriate soft threshold was selected to be *β* = 6 (Figure S2A), and modules were merged based on a threshold value of 0.25. Finally, 18 non‐gray modules were identified (Figure S2B,C). The most negative extent of correlation with the subtypes (*r* = −0.6, *p* = 2E‐16) was observed for the black module, and the most positive extent of correlation with the subtypes (*R* = 0.61, *p* = 1E‐16) was observed for the brown module (Figure S2D). The genes associated with the black and the brown modules were subjected to Cox regression analysis, and 40 prognostic‐related genes (12 protective factors and 28 risk factors) were identified according to the threshold of *p* < 0.05 (Figure S2E). The detailed data are provided in Table S3. The LASSO Cox regression model was then used to identify the potential prognostic markers. The 10‐fold cross‐validation method was used to address the problem of overfitting, and a total of 100 iterations were performed to select the most robust model. The model containing 22 genes was found to be the most robust (Figure 2A). The risk model for 22 genes was constructed based on the optimal λ value of 0.02875 (Figure S3A,B), and the risk score was calculated as follows:

$$\sum i\;\text{Coefficient}\;\left(\text{mRNAi}\right)\times \text{Expression}\;\left(\text{mRNAi}\right)$$

**FIGURE 3:**
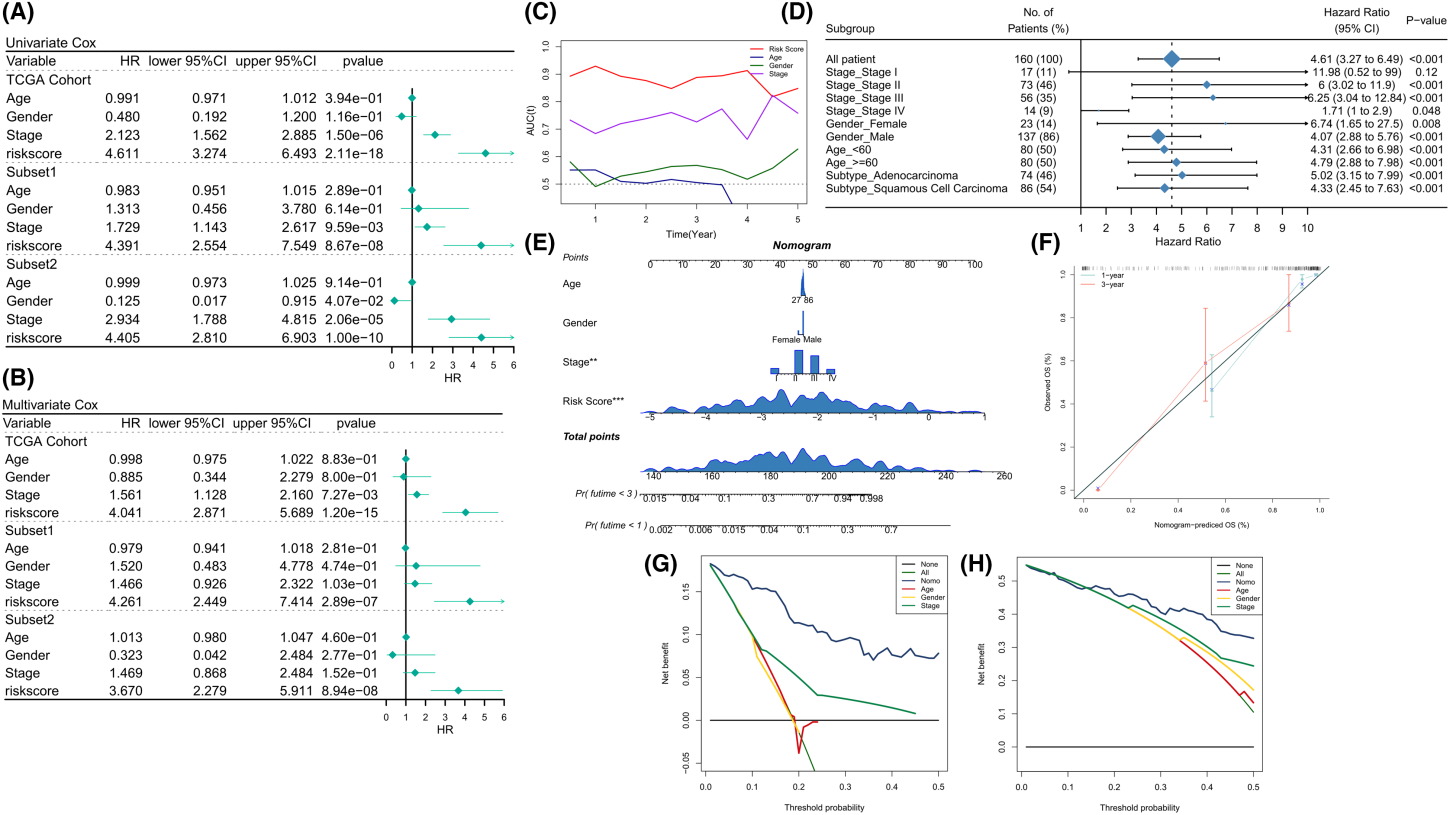
Evaluation and validation of the prognostic value of risk score. (A, B) Forest plots presenting univariate (A) and (B) multivariable regression analyses of clinical characteristics and risk scores as independent prognostic factors. (C) Time‐dependent AUC values of clinical characteristics and risk score (TCGA cohort). (D) Forest map presenting the clinical subgroup univariate COX analysis of risk score in the TCGA cohort. (E) Nomogram specifically quantified the risk assessment of patients based on clinical characteristics and risk score. (F) Nomogram calibration curves at Year 1 and Year 3.Immune landscape and functional enrichment analysis of (G, H) Decision curves presenting the decision efficacy of the Nomogram models and clinical features at (G) Year 1 and (H) Year 3.

The Lasso coefficients of model genes are presented in Table S4. The patients were divided into two groups (high‐risk and low‐risk), based on the median, and the heat map indicated the transcription map of model genes in the high‐risk and low‐risk subgroups (Figure S3C). The risk model exhibited a satisfactory predictive efficacy for the whole training set and two subgroups according to the C‐index (Whole set: 0.8539, Subset1: 0.8375, Subset2: 0.8671; Figure 2B). Results from survival analysis revealed that the prognosis for the high‐risk group was poor (*p* < 0.0001, Figure 2C), and the survival rate for the high‐risk group was low (Figure 2D). Similar results were obtained for both the subgroups (Figure S4A,B). Analysis of the ROC curve revealed that the AUC values of the prognostic model were 0.93, 0.86, and 0.83 at 1, 3, and 5 years, respectively (Figure 2E). Satisfactory prognostic efficacy was also observed in the cases of the two randomly assigned subgroups (Figure S4C,D). Patients in the high‐risk group had a significantly poorer outcome than those in the low‐risk group. The results were based on a survival study conducted with the two randomly allocated categories (*p* < 0.0001, Figure 3F,G).

### 3.4 Predictive independence of risk models

The univariate Cox regression analysis method was used to determine the association among the clinical characteristics (age, sex, and stage), risk score, and prognosis. It was observed that the two independent risk factors for OS in the training set were AJCC TNM staging (HR = 2.123, *p* < 0.001) and risk score (HR = 4.611, *p* < 0.001), which also exhibited good prognostic value in both the subgroups (Figure 3A). However, only the risk score was an independent risk factor for the subgroups (*p* < 0.001; Figure 3B). According to the results obtained from tROC analysis, the risk score was the most reliable predictor of OS (Figure 3C). Subgroup analysis was also conducted, which revealed that good predictive efficacy was observed in the risk score had in all clinical subgroups except for patients of Stage 1 (*p* < 0.05; Figure 3D). Considering the accurate predictive efficacy of the risk model, a nomogram was constructed to validate the risk assessment and survival probability of EC patients at 1 and 3 years (Figure 3E), after proportional hazard assumption test, we confirmed that the nomogram model suit the proportional hazard assumption. We also estimated the variance inflation factor (VIF), and we found the VIF of each variable was close to 1, which suggested that the multicollinearity of nomogram is not obvious. Finally, analysis of the calibration curve revealed that the nomogram was highly accurate (Figure 3F). Decision curves generated at Year 1 (Figure 3G) and Year 3 (Figure 3H) showed that the nomogram exhibited excellent decision efficiency at most threshold conditions.

### 3.5 Correlation between risk models and immune landscapes

**FIGURE 4:**
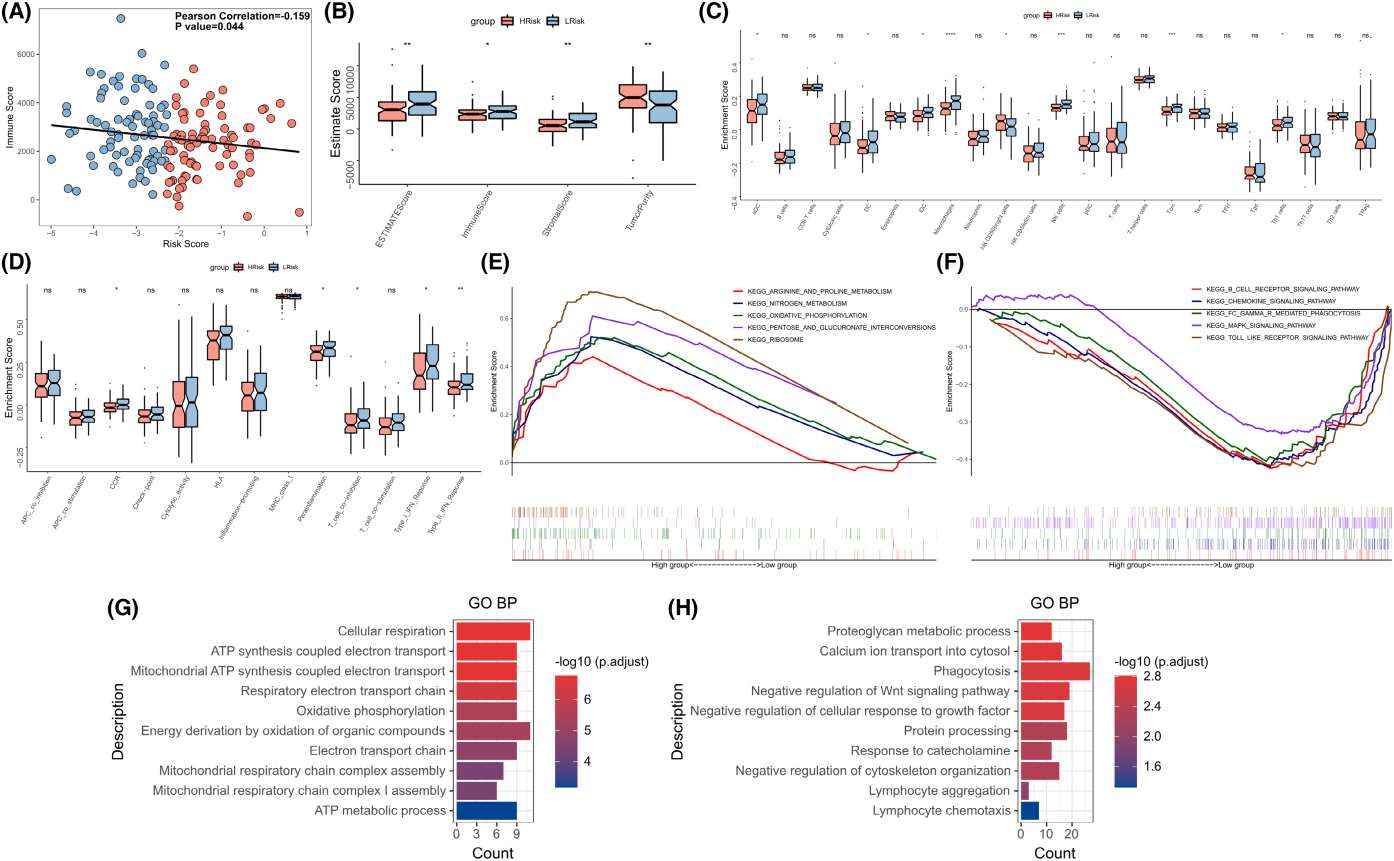
Immune landscape and functional enrichment analysis of high‐ and low‐risk subgroups. (A) Scatter plot presents the negative association between the risk score and immunization score (TCGA cohort) and the Pearson correlation between the risk score and the immunization score. (B–D) Box plots present the (B) ESTIMATE score, (C) proportion of immune cell infiltration, (D) and immune pathway activity for patients with three immune subtypes. *p < 0.05; **p < 0.01; ***p < 0.001; ****p < 0.0001. (E, F) Enrichment plots presenting GSEA enrichment results in (E) high‐risk (F) and low‐risk groups, showing only pathways of interest. (G, H) Bar diagrams presenting the GO enrichment of up‐regulated genes in the (G) high‐risk and (H) low‐risk groups, showing only biological functions of interest.

Immune activity and tumor purity in EC patients were determined using the Estimate algorithm. Results from correlation analysis revealed that the risk score was inversely associated with the immune score (Pearson correlation: *R* = −0.159, *p* = 0.044; Figure 4A). Results from box plot analysis revealed that the immune and Estimate scores for the high‐risk group were low, and the stroma and tumor purity scores were high (*p* < 0.05; Figure 4B). Results from ssGSEA analysis revealed that the infiltration degrees of aDC, DC, iDC, macrophages, NK cells, Tcm, and Th1 cells in the high‐risk group were low, and the infiltration degree of the NK CD56 bright cells was high (*p* < 0.05; Figure 4C). The activities corresponding to IFN response, CCR pathway, Parainflammation, and T cell co‐inhibition were significantly higher in the low‐risk groups (*p* < 0.05; Figure 4D). The ribosome pathway and the oxidative ph osphorylation pathway were found to be prominent in the high‐risk EC patients, whereas the chemokine signaling pathway and MAPK signaling pathway were found to be prevalent in the low‐risk EC patients. Moreover, the Toll‐like receptor signaling pathway and B cell receptor signaling pathway were enriched in the low‐risk group (*p* < 0.05, Figure 4E,F; Table S5), as revealed by the Gene Set Enrichment Analysis (GSEA) method. A difference analysis method was used to further investigate the variations in the biological functions between the high‐ and low‐risk subgroups. The specific findings are presented in Table S6. Results obtained using the GO enrichment analysis method revealed that the up‐regulated genes in the high‐risk group were primarily upregulated during the process of cellular respiration and ATP‐related functions, and the up‐regulated genes in the low‐risk group were primarily linked to phagocytosis, protein processing, lymphocyte chemotaxis, and aggregation (FDR <0.05; Figure 4G,H; Table S7). These results suggested that the tumor proliferation was promoted in the high‐risk group, and the low‐risk group was potentially immune‐activated.

### 3.6 Correlation between the risk scores and somatic variation

**FIGURE 5:**
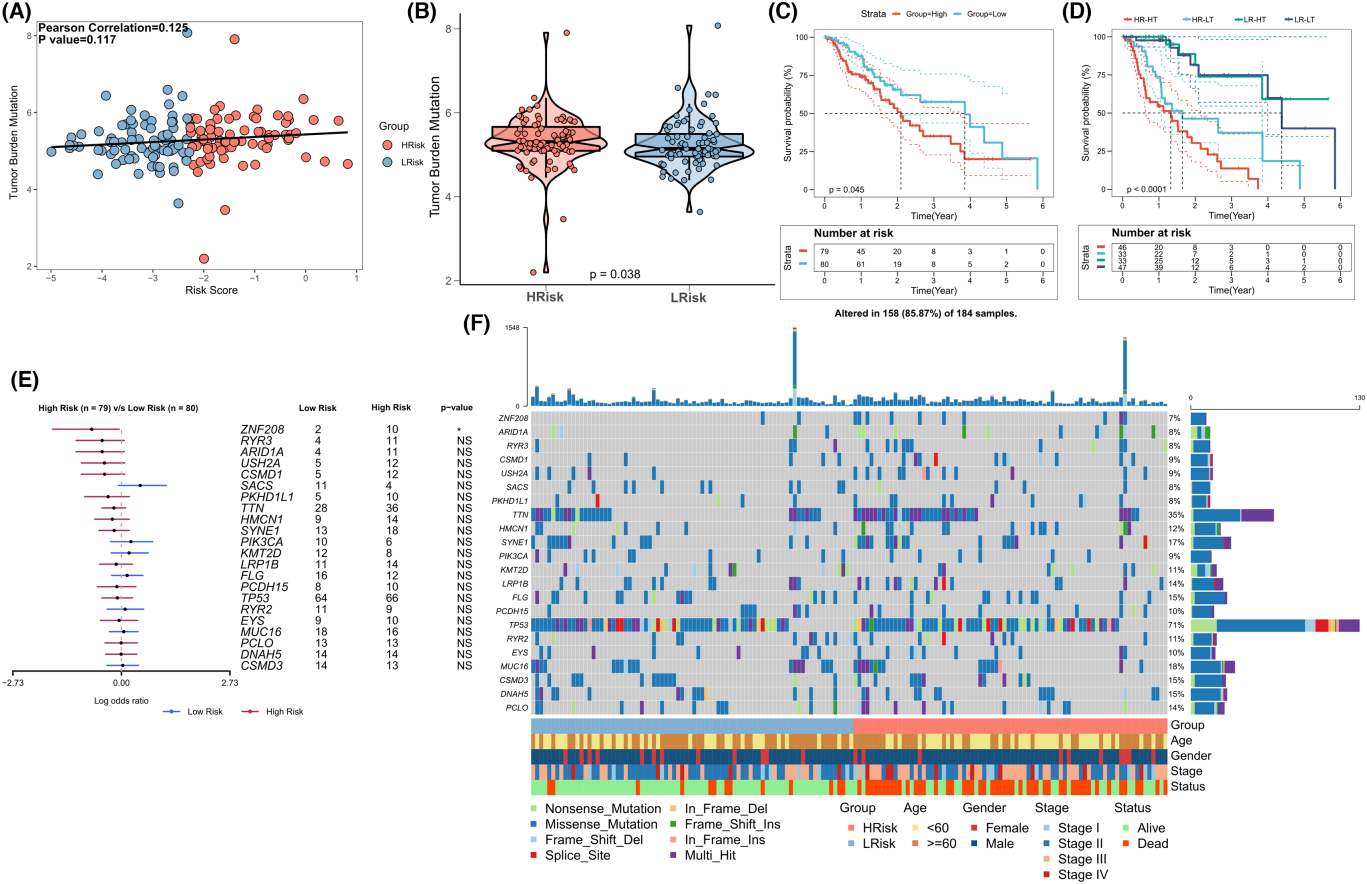
Tumor mutation status of high‐ and low‐risk subgroups. (A) Scatter plot presenting the trend of a positive association between the risk score and mutation (TCGA cohort) and the Pearson correlation between the risk score and mutation burden. (B) TMB difference between high‐ and low‐risk score subgroups. Wilcoxon test, *p* = 0.038. (C) Kaplan–Meier curves corresponding to high‐ and low‐TMB groups (TCGA cohort). Logarithmic rank test, *p* = 0.045. (D) Kaplan–Meier curves corresponding to the members belonging to the TCGA cohort and stratified by TMB and risk scores. Logarithmic rank test, *p* < 0.00011. (E) Forest maps presenting the differences in the mutations of genes with >10 mutations in the high‐ and low‐risk groups. (F) Oncoplot presents the mutation landscape of genes with >10 mutations in the high‐risk and low‐risk groups.

It is well known that tumor mutation burden (TMB) is a powerful prognostic biomarker, but its prognostic effect is heterogeneous among different tumors.^41^, ^42^, ^43^ Several researchers have reported that TMB is associated with immunotherapy responses. This can be attributed to the fact that mutation‐derived antigens and peptides containing neoantigens, recognized and targeted by the immune system, are produced under these conditions to improve the extent to which anti‐tumor immunity is improved.^44^, ^45^, ^46^ Given the clinical significance of TMB, the relationship between TMB and risk score was investigated. Results from correlation analysis revealed a positive correlation between TMB and risk score. Statistical significance (Pearson correlation: *R* = 0.125, *p* = 0.117; Figure 5A) was not observed. Results from Box‐plot revealed that the levels of TMB in high‐risk patients were higher than the levels in low‐risk patients (Wilcoxon test; *p* = 0.038; Figure 5B). Results obtained by conducting survival analysis revealed that patients belonging to the TCGA‐EC cohort and characterized by high TMB exhibited a low OS score (*p* = 0.045; Figure 5C). The synergies in the prognostic stratification of these two biomarkers were evaluated considering the good predictive efficacy of the risk score. TMB did not affect the predictions based on the risk score, according to stratified survival analysis. The risk subtypes exhibited a significant difference among the survival rates between the high and low TMB subgroups (log‐rank test, *p* = 0.002; High TMB & High Risk Score (HR‐HT) versus High TMB & Low Risk Score (LR‐HT), *p* < 0.001; Low TMB & High Risk Score (HR‐LT) versus Low TMB & Low Risk Score (LR‐LT), *p* < 0.001; Figure 5D). These data indicated that the risk score might be a viable predictor (independent of TMB) and could be used efficiently for the prediction of patient outcomes. In addition, the mutation frequency of highly mutated genes in both groups was compared. Analysis of the Forestplot revealed that the mutation frequency of ZNF208 was significantly higher (*p* < 0.05) in the high‐risk group than that in the low‐risk group. Although the high‐risk group was characterized by a large number of mutations in most genes, statistical difference was not observed (*p* > 0.05; Figure 5E). The mutation landscape of the high‐mutation genes in the EC patients belonging to the high‐low risk subgroup is presented in detail in Figure 5F.

### 3.7 Risk score can be used as a predictor of immunotherapy and chemotherapy

**FIGURE 6:**
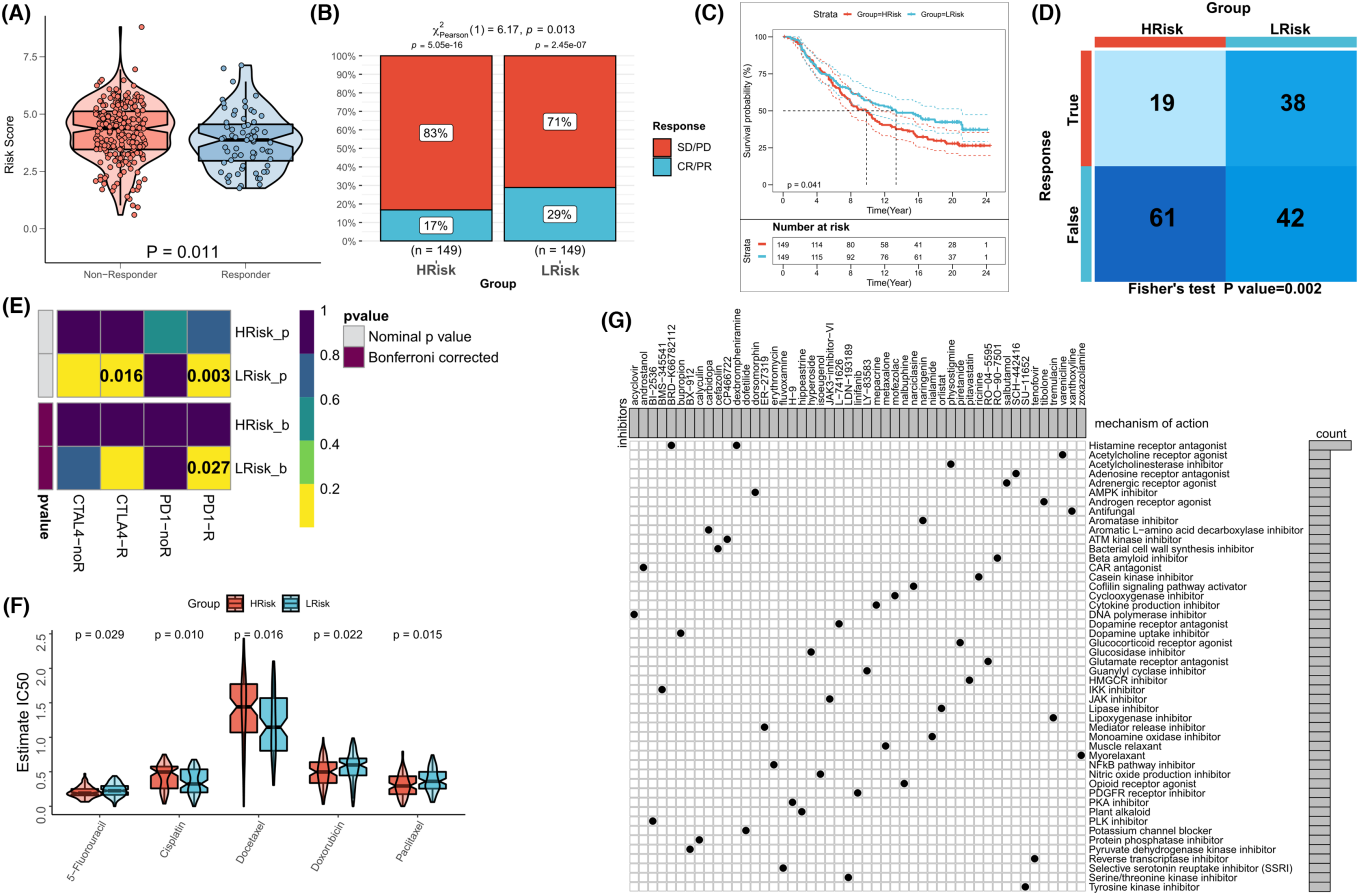
Risk score predicted the benefit of treatment. (A) IMvigor210 risk score with various clinical response levels against PD‐L1. Wilcoxon test, *p* = 0.011. (B) Rates of clinical response toward INMVIGOR 210 (immunotherapy) under conditions of high or low risk (score [CR]/partial response [PR] and stable [SD]/progressive disease [PD]). (C) Kaplan–Meier curves corresponding to the IMvigor210 cohort for high and low‐risk patients. Test of the rank of the logarithm, *p* = 0.041. (D) Results obtained using the TIDE algorithm, revealing a greater immuno‐response, Chi‐square, *p* = 0.002 in patients with a low‐risk score. (E) Subclass mapping revealed that low‐risk score patients were more responsive toward anti‐PD1 therapy (FDR = 0.027), with a *p*‐value being <0.05 (F) Box plot presents the sensitivity of the high‐ and low‐risk groups (TCGA cohort) toward the five widely used EC drugs. (G) Small molecule drugs as EC treatment candidates (based on risk models). Rows represent small molecule inhibitors, and columns represent biological pathways that the small molecule inhibitors target.

The method of cancer immunotherapy has revolutionized the field of treatment of various tumor types. Attention was paid to therapeutic methods involving immune checkpoint inhibitors (ICI), such as anti‐programmed death 1 (anti‐PD‐1), anti‐ligands of PD‐1 (anti‐PD‐L1), and anti‐cytotoxic T lymphocyte antigen 4 (anti‐CTLA4).^43^, ^47^, ^48^ Furthermore, analyses were conducted to examine the utility of risk scores in predicting the benefits of immunotherapy. The previously presented formula was used to categorize the patients belonging to the IMvigor210 cohort, treated with anti‐PD‐L1, into high and low‐risk categories. The box plot was analyzed, and the results revealed that significantly high‐risk scores were recorded for the patients who did not react to immunotherapy (Wilcoxon test, *p* = 0.011; Figure 6A). The low‐risk group was characterized by a high objective response rate toward anti‐PD‐L1 therapy. The degree of response realized in this case was higher than that realized in the high‐risk group (Chi‐square test, *p* = 0.013; Figure 6B). In addition, patients in the high‐risk group exhibited a shorter survival time (log‐rank test, *p* = 0.041, Figure 6C) than the patients in the low‐risk group. The response of the patients toward anti‐PD1 and anti‐CTLA4 therapy was assessed using the TIDE algorithm, and the patients in the low‐risk group showed a higher response rate toward immunotherapy (Chi‐square test *p* = 0.002; Figure 6D) than the patients in the high‐risk group. Subclass Mapping results revealed that the low‐risk group was more sensitive toward anti‐PD1 treatment (FDR = 0.027) and tended to be more sensitive toward CTLA4 treatment (*p* = 0.016; Figure 6E) than the high‐risk group. In addition, the sensitivity to chemotherapy was checked, and it was found that patients in the high‐risk group were more sensitive to 5‐fluorouracil, doxorubicin, and paclitaxel. In contrast, patients in the low‐risk group were more sensitive to cisplatin and docetaxel (*p* < 0.05; Figure 6F). Differential genes in both groups were identified as possible small molecule drug targets, and 49 potential small molecule drugs were identified by submitting the differential genes to the Clue database. These small molecule drugs and the 48 biological pathways they target are presented in the Waterfall diagram (Figure 6G).

## 4 DISCUSSION

EC is one of the most aggressive malignancies and is also a major cause of cancer‐related mortality worldwide.^49^ Although diagnoses and treatment methods have been significantly improved over the years, the 5‐year OS rate of patients suffering from EC remains low.^1^ The establishment of new therapeutic modalities in EC is a hot topic in the field, and it has been observed that the multi‐mode combination therapy method presents good prospects. A combination of immunotherapy with surgical methods, chemotherapy, and RT can be potentially used for the development of effective treatment methods.^50^ However, as there are serious side effects of using the methods of combination therapy, the techniques and methods used for immunotherapy need to be studied in detail.^50^ Even though several biomarkers have been identified for the predictive factors of immunotherapy prognosis, the efficiency is not satisfactory to date.^51^ Genomic changes of IRG in EC should be studied in detail to understand EC tumorigenesis, development, and complex TME. Classification schemes based on IRG characteristics may provide new insights for predicting patient prognosis and developing new individualized treatment strategies.

We focused on genomic changes in the IRGs of EC patients. The experiments were conducted before and after subjecting the patients to conditions of neo‐chemoradiotherapy. We also analyzed classical TCGA‐EC datasets based on significantly altered IRGs. Based on these IRGs, the patients were divided into three different immune subtypes. The results suggested that high levels of immune‐activated phenotypes (Clusters 2 and 3) were linked to antigen presentation, cellular immunity, and antitumor immunity. The high levels were also associated with favorable prognosis, whereas immune failure phenotypes (Cluster 1) associated with high tumor purity and low immunoactivity were associated with poor prognosis. The results agreed well with the results presented in the literature.^52^, ^53^ Given the interesting differences between the immune subtypes, it was hypothesized that risk models based on genes associated with immune subtypes could be used for developing novel patient‐specific therapeutic strategies.

Several researchers have reported that TME‐based immune characteristics can be effectively used for the prediction of tumor prognosis and immunotherapy decision‐making.^54^, ^55^, ^56^ Highly accurate results could be obtained. We used the lasso model to build robust immune characteristic risk models. Considering the randomness of the lasso regression cross‐validation method, 100 iterations were performed to obtain the most stable model. Results from survival analysis and time‐dependent AUC and the C index were used to validate the high efficacy of the risk scores in predicting OS of patients suffering from EC. Results obtained using the stratified subgroup analysis method suggested that the risk score might help predict the survival outcomes in individuals characterized by varying clinical traits. mmune characteristics have emerged as the new tools that can be used for understanding the incidence and development of tumors and TME. The results presented herein revealed that the high‐risk group exhibited higher activities corresponding to cellular respiration and ribosomal‐related pathways than the low‐risk groups, while the low‐risk group exhibited higher activities corresponding to immune‐related pathways and protein processing pathways than the high‐risk groups. These suggested that the high‐risk score might be used to predict the tumor metabolic activity, while the low‐risk score might be used to predict the high immune activity. The subgroups of patients with TME were studied, and the results revealed that the infiltration degrees corresponding to aDC, DC, iDC, macrophages, NK cells, Tcm, and Th1 cells in the high‐risk group were low. The IFN response and the activity of the CCR pathway of Type 1 and Type 2 groups were also low. Patients belonging to the high‐risk group were believed to be characterized by an immune failure phenotype, whereas the patients in the low‐risk group were believed to exhibit a favorable immune activation phenotype. The results obtained using ESTIMATE also confirmed a negative correlation between the risk score and the immune score (*r* = −0.159). A higher degree of tumor purity was observed in the high‐risk group (compared to the low‐risk group), and higher immune activity was observed in the low‐risk group (compared to the high‐risk group). TME differences may result in poor outcomes in high‐risk subgroups. This result agreed well with the results reported previously.^52^, ^53^

TMB is considered a biomarker for immunotherapy response and prognosis of tumor patients.^41^, ^42^, ^43^ In general, higher TMB predicts a higher benefit of immunotherapy. Heterogeneity in prognostic effects across different tumors is observed. TMB was higher in the high‐risk group (compared to the low‐risk group), and the most‐mutated genes were characterized by increased mutation frequency in the high‐risk group. However, as noted above, patients in the high‐risk group exhibited lower immune activity than the patients in the low‐risk group, suggesting that a high level of TMB does not always indicate high immunogenicity. Results obtained by conducting stratified survival analysis revealed that the risk score could potentially predict the OS of patients and the results were independent of TMB. Although ZNF208 was the only significantly different mutation gene between the two risk groups, there was no evidence that ZNF208 was associated with the progression of cancer.

Recently, several clinical studies have confirmed the encouraging efficacy of immunotherapy in EC, especially anti‐PD‐1 therapy.^57^, ^58^, ^59^ However, like most other solid tumors, only a small percentage (20%–30%) of patients with EC benefit from anti‐PD‐1 therapy.^57^, ^58^, ^59^ Differences in the efficacy of immunotherapy are closely related to the heterogeneity of the individual immune microenvironment of each patient.^60^ Previous results have suggested that different TME phenotypes are associated with the efficacy of chemotherapy, targeted therapy, and immunotherapy methods.^61^ Therefore, a combination of chemotherapy, targeted therapy, and immunotherapy should be used and the method optimized (based on an immunological characteristic risk model) to obtain better results. Multiple algorithms were used to explore the decision‐making effect of the risk models for anti‐PD1, anti‐PD‐L1, and anti‐CTLA‐4 treatments. Results obtained using the TIDE algorithm and those obtained by conducting the subclass mapping analysis indicated that the low‐risk group might be more sensitive toward anti‐PD1 therapy than the high‐risk group. Furthermore, 298 patients treated with anti‐PD‐L1 in the IMvigor210 cohort were screened, and the results revealed that the low‐score patients exhibited improved survival and response rates toward anti‐PD‐L1 therapy (compared to high‐score patients). In addition, results from drug sensitivity analysis indicated that the risk model might be useful for planning the chemotherapy‐related treatment methods. The response rates of conventional drugs, such as cisplatin, docetaxel, doxorubicin, paclitaxel, and 5‐fluorouracil (used to treat EC) were different in high‐ and low‐risk subgroups. These results revealed that the immune features influenced the efficiency of chemotherapy and the targeted treatment method. They also influenced immunotherapy response. In addition, 49 potential small molecule drugs were identified based on the differential genes of the two subgroups.

We first proposed an immune‐related risk model (based on the genomic changes occurring under conditions of RT), which could accurately predict the OS in patients with EC. Several researchers have previously proposed immune‐related risk models for EC. Our model exhibited better accuracy than the previously reported methods, and it was revealed by the results obtained by analyzing the ROC curve.^56^, ^62^, ^63^ Moreover, our model could also be used to classify “cold” and “hot” tumors, which results in differences in sensitivity toward immunotherapy and chemotherapy. These results suggested that our model exhibited great promise and could be used for multimodal combination therapy to treat EC.

Even though our model exhibited excellent predictive efficacy in the TCGA‐EC cohort, the results should be further verified. Clinicopathological variables should not be overlooked. Unfortunately, as there is a lack of detailed EC sequencing and follow‐up data, we have no additional external cohort to validate the model. We can only test the findings using a randomly assigned subgroup validation cohort. Moreover, our study only considered the heterogeneity between patients, not intratumoral heterogeneity. Although we determined the predictive performance of the model for immunotherapy response using multiple algorithms, clinical trials should be conducted in a larger cohort of EC under conditions of immunotherapy to confirm the effectiveness of the model for clinical evaluation and decision making. Finally, further in vivo and in vitro experiments should be conducted to study the functional differences and specific regulatory mechanisms of TME among the different subtypes.

In conclusion, an immune characteristic risk model to predict the OS of patients suffering from EC was constructed, and the model exhibited good predictive efficacy. The risk score was found to be associated with tumor metabolism and immune response and could be used to identify the ideal candidates for individualized immunotherapy and RT.

## AUTHOR CONTRIBUTION

Xiangyu Su designed the concept and experiments; Chenchun Fu prepared the manuscript draft; Shicheng Feng and Sheng Wang collected the data and did the analysis; Xiangyu Su revised the manuscript. All the authors approved the final proof.

## CONFLICT OF INTEREST

All authors declare that there is no potential conflict of interest.

## ETHICS AND CONSENT STATEMENT

Not applicable.

## PUBLICATION PERMISSION

All authors have agreed to publish this paper.

## Supporting information


Figure S1

Figure S2

Figure S3

Figure S4

Table S1‐S7

## Data Availability

This research is based on a publicly available dataset. Detailed data can be obtained by contacting the corresponding author.
