# Supplementary figures and images for: Development and validation of a prognostic model for esophageal carcinoma based on immune microenvironment using system bioinformatics
Source: Cancer Med. 2022 Jun 30;12(2):2089–103. doi: 10.1002/cam4.4985 (PMC9883539; doi:10.1002/cam4.4985)

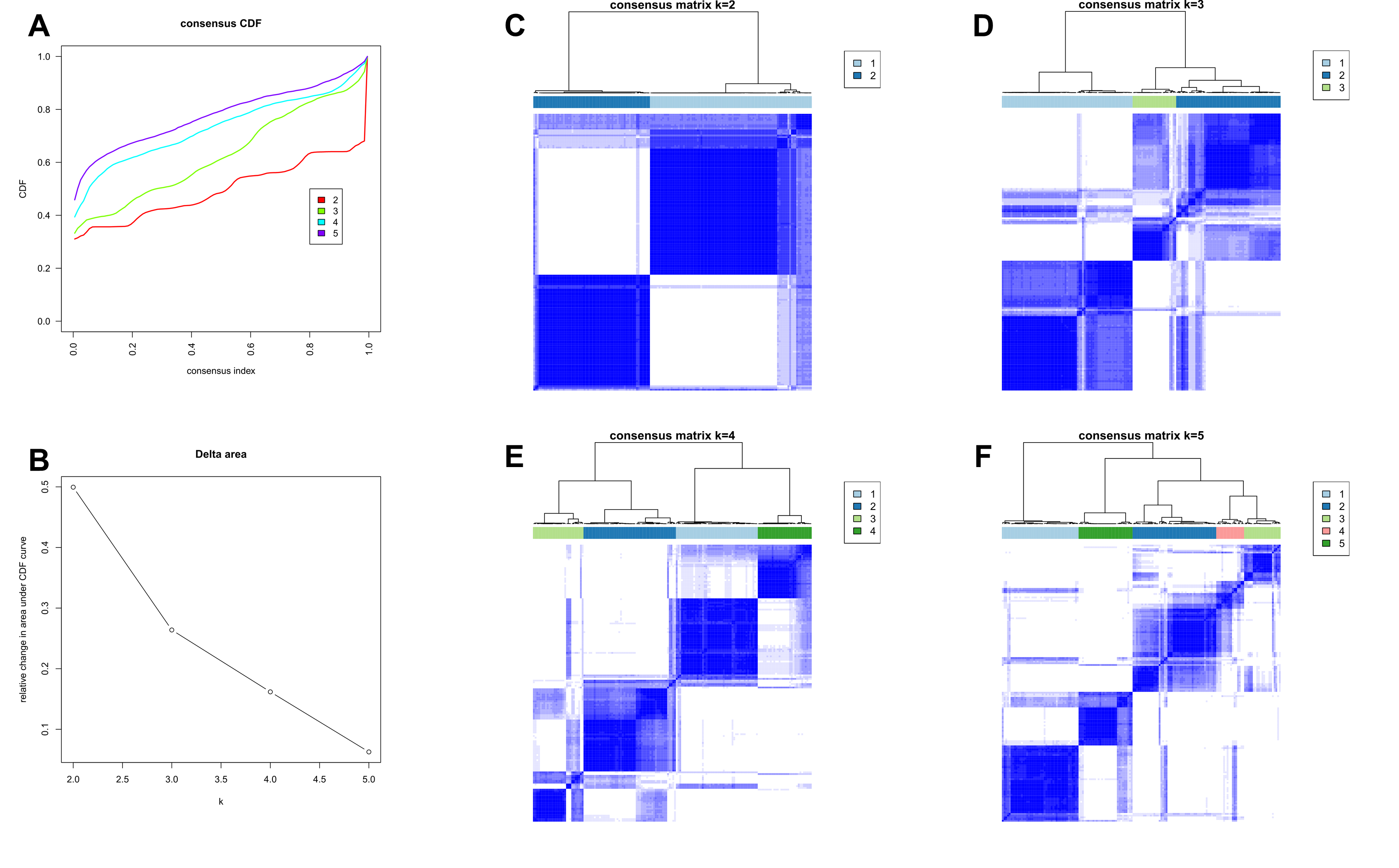

Supplement: Supplementary file 1 — Figure S1 [file CAM4-12-2089-s005.tif]

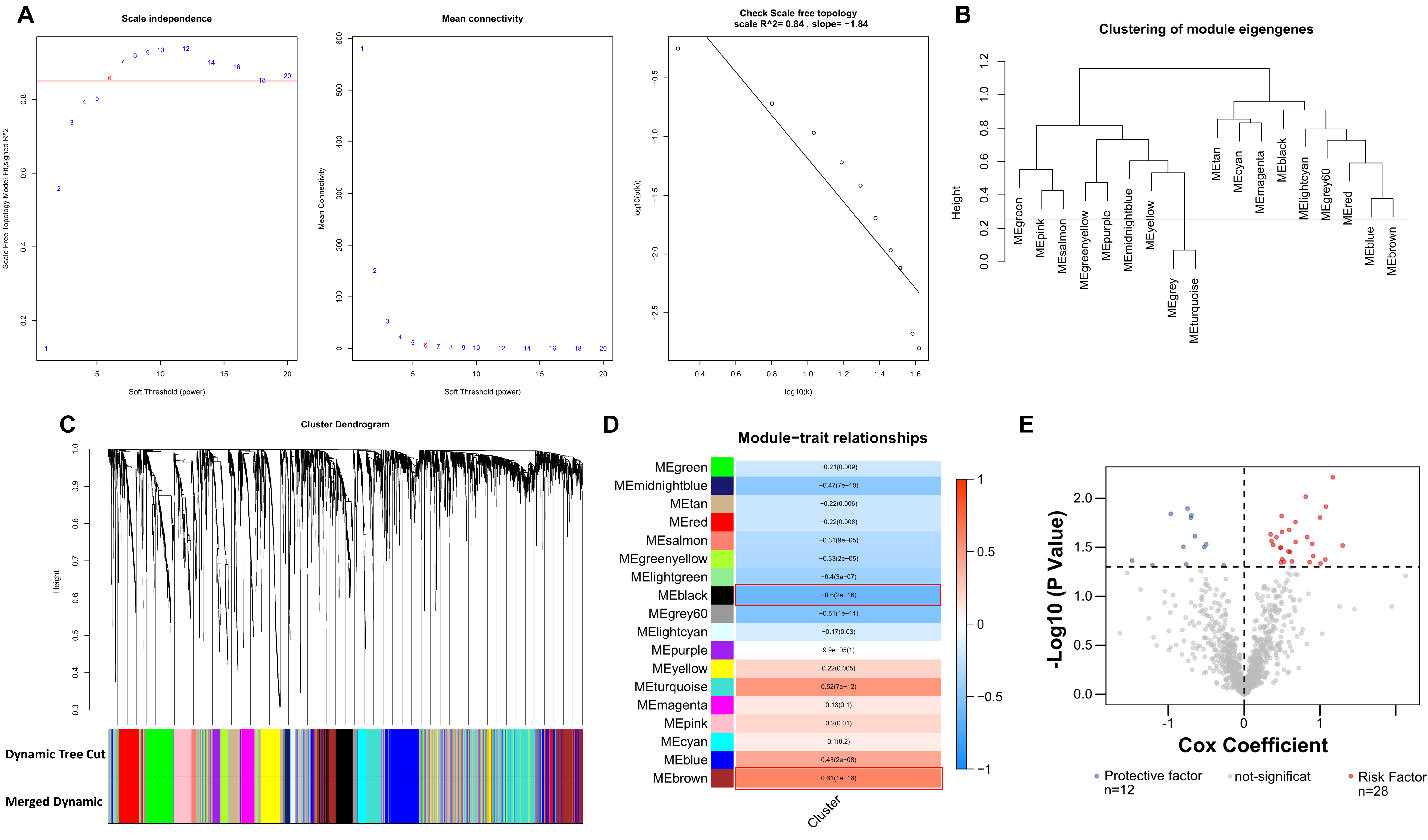

Supplement: Supplementary file 2 — Figure S2 [file CAM4-12-2089-s002.tif]

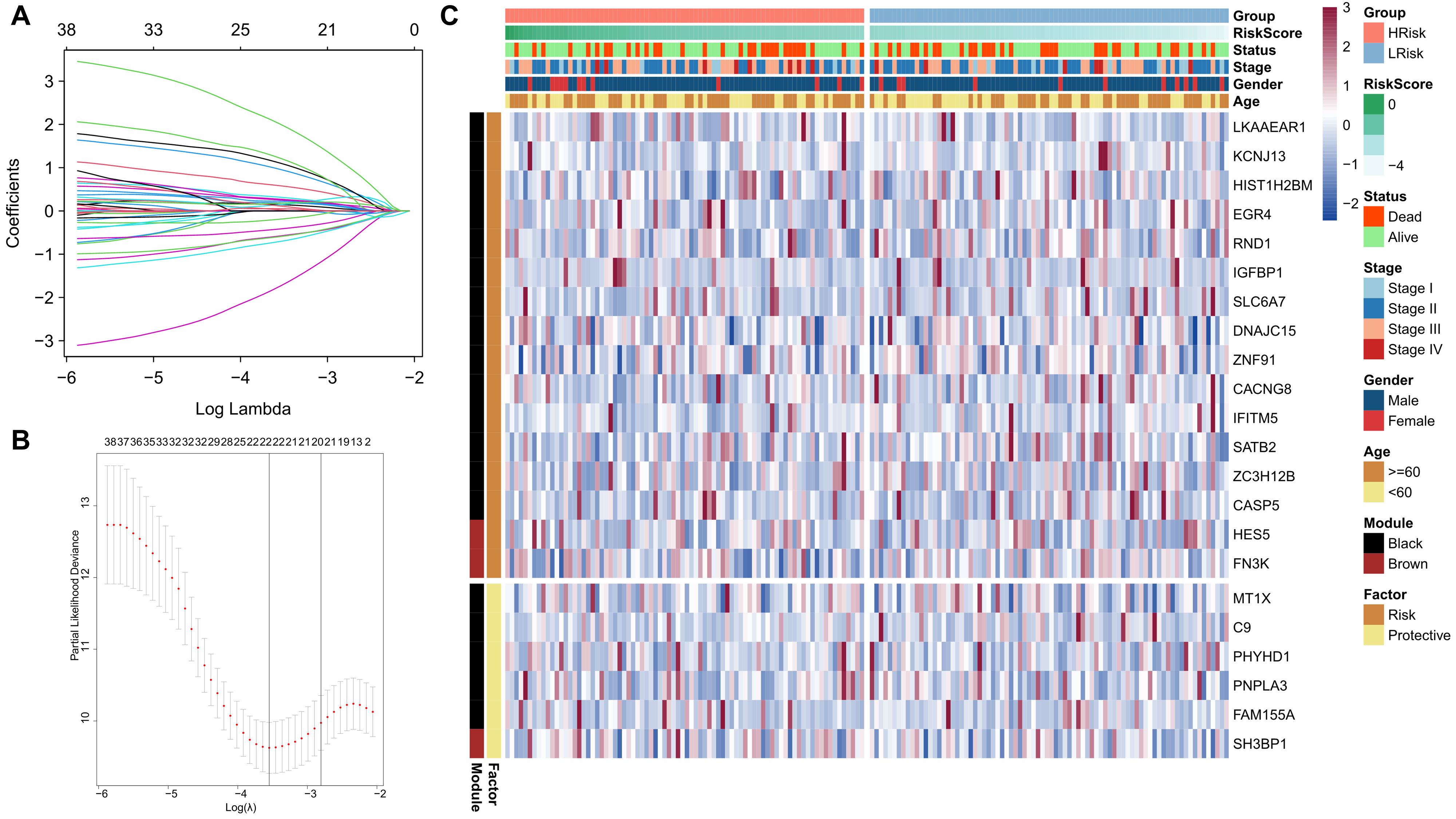

Supplement: Supplementary file 3 — Figure S3 [file CAM4-12-2089-s004.tif]

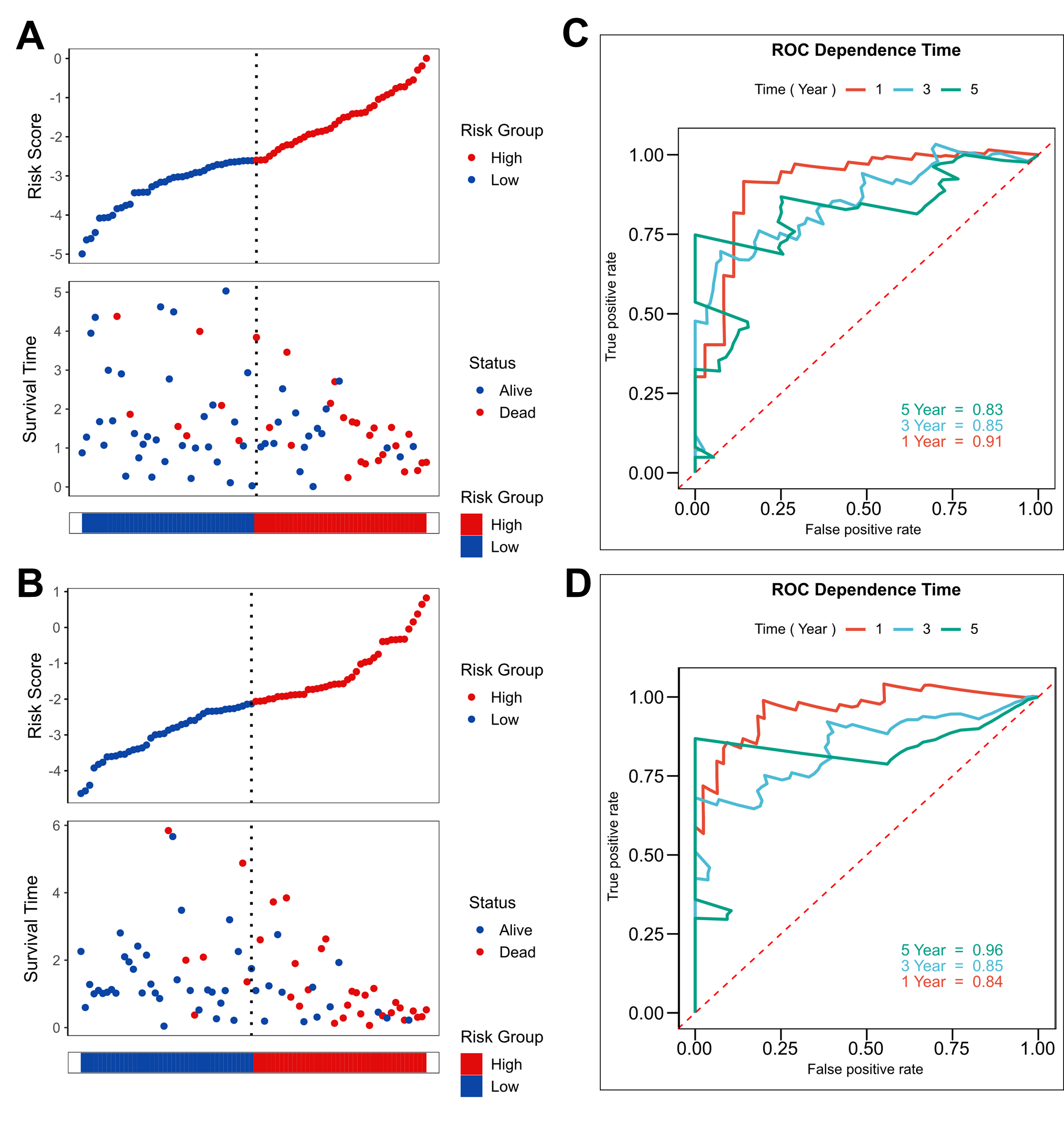

Supplement: Supplementary file 4 — Figure S4 [file CAM4-12-2089-s001.tif]
